# Cardioprotective Effects of Dietary Phytochemicals on Oxidative Stress in Heart Failure by a Sex-Gender-Oriented Point of View

**DOI:** 10.1155/2020/2176728

**Published:** 2020-01-06

**Authors:** Klara Komici, Valeria Conti, Sergio Davinelli, Leonardo Bencivenga, Giuseppe Rengo, Amelia Filippelli, Nicola Ferrara, Graziamaria Corbi

**Affiliations:** ^1^Department of Medicine and Health Sciences, University of Molise, Via Francesco De Sanctis, 1, 86100 Campobasso, Italy; ^2^Department of Medicine, Surgery and Dentistry “Scuola Medica Salernitana”, University of Salerno, Via S. Allende, Baronissi, 84081 Salerno, Italy; ^3^Department of Translational Medical Sciences, University of Naples Federico II, Via Pansini, 5 -, 80138 Naples, Italy; ^4^Istituti Clinici Scientifici Maugeri SpA Società Benefit” (ICS Maugeri SpA SB), Via Bagni Vecchi, 1, 82037 Telese Terme (BN), Italy

## Abstract

Dietary phytochemicals are considered an innovative strategy that helps to reduce cardiovascular risk factors. Some phytochemicals have been shown to play a beneficial role in lipid metabolism, to improve endothelial function and to modify oxidative stress pathways in experimental and clinical models of cardiovascular impairment. Importantly, investigation on phytochemical effect on cardiac remodeling appears to be promising. Nowadays, drug therapy and implantation of devices have demonstrated to ameliorate survival. Of interest, sex-gender seems to influence the response to HF canonical therapies. In fact, starting by the evidence of the feminization of world population and the scarce efficacy and safety of the traditional drugs in women, the search of alternative therapeutic tools has become mandatory. The aim of this review is to summarize the possible role of dietary phytochemicals in HF therapy and the evidence of a different sex-gender-oriented response.

## 1. Introduction

Despite recent advances in clinical/diagnostic tools and therapies, the incidence and prevalence of heart failure (HF) show a steady increase [1]. From the pathophysiological point of view, this condition is associated with chronic overactivation of the adrenergic system [2–4], endothelial dysfunction, increased oxidative stress [5], and mitochondrial dysfunction; all these abnormalities are involved in the development and the progression of HF [6]. Nowadays, although drug therapy and the implantation of devices have demonstrated to increase survival, their use is limited by important side effects [7] that worsen the HF patients' quality of life. Moreover, the demographic changes in population composition have recently determined an increasing difficulty in the therapeutic approach of HF. Starting with the evidence of the feminization of the world population and the scarce efficacy and safety of the traditional drugs in women, the search for alternative therapeutic tools has become mandatory. Despite sex referring to biological factors and gender to psychosocial, cultural, and environmental factors, it is difficult to separate one from each other. Both are multidimensional, entangled, and interactive factors that may influence the pharmacological response. Then, it has been suggested that the simultaneous use of sex-gender terms is more appropriate [8]. From this view, the dietary phytochemicals appear, thanks to their safety and efficacy, the ideal candidate for the HF therapy in women. The aim of this review is to summarize the cardioprotective effects of natural products in HF therapy and the evidence of a different sex-gender-oriented response to oxidative stress modulation.

## 2. Sex-Gender and HF

### 2.1. Sex-Gender Differences in HF

The prevalence of HF affects about 2.6 million women and 3.1 million men in the USA [9], with higher prevalence in advanced age. However, women tend to be older than men at diagnosis and exhibits a different phenotype: the HF with preserved ejection fraction (HFpEF) is more frequent in women and new onset of HF with reduced ejection fraction (HFrEF) in men [10]. Moreover, despite women with present HF at an older age, where comorbidities are more frequent, some studies showed that women have a lower cardiovascular and all-cause mortality [11, 12], suggesting that the phenotypic differences in HF presentation and prognosis between women and men may be the consequence of progressive, sex-specific changes in cardiac and vascular physiology. Furthermore, the menopausal transition may influence the development of cardiovascular risk factors in women [13]. Women demonstrate greater, body-size-adjusted increases in the left ventricle (LV) wall thickness and concentric remodeling than men, which predispose to myocardial stiffness and diastolic dysfunction. Furthermore, the age-related increase in left ventricle ejection fraction (LVEF) is more pronounced in women [14]. Animal models indicate that female rats are more likely to develop concentric myocardial hypertrophy [15]. These differences in LV remodeling are consistent with the highest likelihood of women to present HFpEF. On the other hand, men are more likely to show age-related increases in the LV cavity dimension and LV systolic dysfunction, which are hallmarks of HFrEF [14]. Consistent with these observations, male rats generally develop eccentric myocardial hypertrophy and fibrosis [15]. These different underlying processes could be responsible for the potential differences in drug tolerance and toxicity [16] to therapeutic interventions in women. Indeed, the most common therapeutic modalities for HF currently include therapies effective in the treatment of HFrEF; conversely, their efficacy in the treatment of HFpEF, the most common HF type in women, has been largely inconclusive [17]. Then, the understanding of the differences in pathophysiological response requires also the study of the different molecular pathways implicated in the different phenotypes of HF.

While HFrEF is linked with ischemia and cardiomyocyte loss [18], HFpEF is associated with advanced age [19], obesity [20], and hypertension [21]. These comorbidities induce the extensive myocardial expression of endothelial adhesion molecules [22]. A progressive reduction in the capillary density (i.e., microvascular rarefaction) in HFpEF hearts [23] activates a transforming growth factor-*β*- (TGF*β*-) signaling cascade that is profibrotic and leads to myocardial collagen accumulation [24, 25].

### 2.2. Role of Oxidative Stress in HF

Activation of the sympathetic nervous system and the renin-angiotensin-aldosterone axis in HF is associated with oxidative stress, and it is already well-established that, in the genesis of HF, reactive oxygen species (ROS) production within the myocardium and the vasculature is substantially increased [5, 26, 27]. Oxidative stress is defined as an imbalance between the production of ROS and the endogenous antioxidants defense system [28, 29].

ROS are high reactivity oxygen-based chemical species that include free radicals, such as superoxide (O2^−^), hydroxyl radical (OH), and nonradical molecules capable of generating free radicals, such as hydrogen peroxide (H2O2). The role of the antioxidant defense system is to scavenge and degrade ROS to nontoxic molecules. Well-known enzymes with antioxidant properties are superoxide dismutase (SOD), glutathione peroxidase, and catalase [30]. Mitochondria, nicotinamide adenine dinucleotide phosphate (NADPH) oxidases (NOXs), and uncoupled NO synthase (NOS) are considered relevant sources of ROS generation during HF development. Under physiological conditions, NADH and FADH2 generation is required for sequential redox reactions in mitochondria and ATP production, and minimal quantity of ROS is produced. In HF models, increased generation of O2^−^ , hydroxyl radicals, and H2O2 has been identified an [31, 32]. Moreover, the myocardial expression of ROS correlates with left ventricular contractile dysfunction. Although the majority of experimental models are performed on mice or rats following myocardial infarction induction [33], data from clinical studies in patients with ischemic cardiomyopathy also confirmed the relationship between increased levels of ROS and left ventricular dysfunction [34]. In fact, in conditions such as hypoxia or ischemia where oxygen availability is reduced, mitochondrial generation of ROS is enhanced and this can contribute to cardiomyocyte damage [28, 35]. In addition, increased ROS levels influence mitochondrial DNA damage which may lead to additional mitochondrial ROS generation [36]. Ca^2+^ signaling alternations are other hallmarks for HF, and systolic Ca^2+^ transient amplitude is reduced with a slower rate-of-rise. Consequences of these modifications are decreased cardiomyocyte shortening, slower contractile kinetics, and delayed relaxation. At the molecular level, these findings are explained by an increased leak of Ca^2+^ from the sarcoplasmic reticulum via ryanodine receptors, downregulation of sarcoplasmic reticulum ATPase (SERCA2A) together with upregulation of Na/Ca^2+^ exchanger [37]. These perturbations at the mitochondrial level affect the NADH and NADPH activity, enhancing the prooxidative state and increasing mitochondrial ROS production [38].

NADPH oxidase has been reported as a major source of ROS generation in HF [39]. Endothelial cells and activated leukocytes are main sources of NADPH oxidase ROS generation. Different studies over the last decade have tried to investigate the role of specific catalytic units of NADPH oxidase such as Nox2 and Nox4 in HF development and progression [40, 41]. Indeed, genetic deletion of Nox2 in animal models of systolic dysfunction improved left ventricular remodeling. Pressure overload models of HF triggers NOS uncoupling, generation of ROS, and its inhibition-attenuated cardiac dilatory remodeling and reduced ROS level [42]. Of interest, a crosslink between NADPH oxidase activation and mitochondrial dysfunction mediated by Angiotensin II has been also described [43]. Angiotensin II initially activates NADPH oxidase and production of O2^−^ and H2O2, which induces mitochondrial generation of ROS and modulates endothelial NO production.

As previously described, whereas in HF genesis and progression, a prominent role is ascribed to ROS production and accumulation, exhaustion of endogenous antioxidants defense system has been reported in myocardial infarction animal models [43], and inversely, overexpression of antioxidant enzymes such as glutathione peroxidase improves left ventricular remodeling in ischemia/reperfusion HF model [44, 45].

Several murine models of systolic HF have showed reduction of other antioxidative defenses as SOD and NAD [33, 46–49]. Of interest, depleted blood glutathione levels were significantly associated with symptomatic status in patients with ischemic cardiomyopathy [50]. In contrast, in pacing induced HF experimental model, no modification of antioxidant defense enzymes including SOD, glutathione peroxidase, and catalase was described indicating that oxidative stress in this type of HF might be primarily caused by the enhancement of ROS generation rather than the decline in antioxidant defenses [51]. Preclinical studies have revealed the existence of an uncoupled sNO enzyme state where NO release was replaced by O2^−^ production, and inhibition of eNOS resulted in decreased ROS generation [52, 53]. Furthermore, AT1 receptor blockage reduced ROS production, improved NO release, and cardiac remodeling in idiopathic-dilated cardiomyopathy experimental model [54]. Interestingly, in an experimental model of HFpEF, decreased myocardial NO levels lead to increased cytosolic Ca^2+^ and diastolic dysfunction and these effects were restored by supplementation with eNOS cofactor tetrahydrobiopterin [55]. It is believed that in HFrEF, major generation of ROS occurs in cardiomyocytes and this process induces maladaptive remodeling through the activation of cardiomyocyte necrosis, apoptosis, and reactive cardiac fibrosis, while HFpEF, a chronic proinflammatory state induced by comorbidities, is responsible for ROS generation in endothelial cells. Further, this process effects cardiac remodeling by inducing cardiomyocyte hypertrophy and myocardial stiffness.

### 2.3. Sex-Gender Differences in Oxidative Stress

Notably, clinical and experimental data suggested a greater antioxidant potential in females over males [56]. In particular, ROS production is higher in the vascular cells from males than in the cells from females [57, 58], suggesting that women are less susceptible to oxidative stress. Indeed, oxidative stress occurs as a result of an imbalance between ROS production and the antioxidant defense system. Brandes et al. [59] found that, under normal conditions, the male rat aortas generated more superoxide radicals than the female aortas. In contrast, a study [60], comparing coronary artery disease (CAD) in postmenopausal women with males, demonstrated that, even in the control group who did not have CAD, postmenopausal women had higher oxidative stress levels than men, with women with CAD having ROS levels almost three times higher than men with CAD [60]. This suggests the possibility that estrogen lowers oxidative stress in premenopausal females. These findings could explain, at least in part, the different HF phenotype between genders.

A difference in the expression and/or activities of antioxidant enzymes between males and females may also be explained by the different antioxidant response. Regarding superoxide dismutase (SOD), there is no uniform consensus on gender differences. Chen et al. [61] reported no significant difference in SOD activity levels between male and female mice in the heart, while Barp et al. [62] in female rats' heart found higher SOD activity levels than in males. Interestingly, the SOD activity levels in both male and female rats were significantly decreased after castration compared to their respective controls [62], suggesting a possible association between sex hormones and SOD activity levels. It is more likely that males could have higher levels of superoxide production due to NADPH oxidase activity. A study [63] found no gender difference in the vascular response to hydrogen peroxide, thus suggesting that the difference between male and female ROS production could be due to NADPH oxidase activity. In particular, NADPH oxidase subunits were found to exhibit gender discrepancies. Expression of Nox1 and Nox4 was higher in males than in females, suggesting that gender differences in superoxide formation could be due to the activity of these two subunits [63]. Stimuli that activate Nox4 include ischemia, hypoxia, and adrenergic stimuli, all present and enhanced in the setting of HF. The implications of Nox4 for HF are still controversial. Kuroda et al. [41] demonstrated that, in cardiac-specific Nox4 knockout mice, the upregulation of Nox4 was primarily responsible for the increased mitochondrial O2− production in response to pressure overload. Furthermore, Nox4 played a critical role in mediating mitochondrial dysfunction, apoptosis in cardiac myocytes, and eventual LV dysfunction in response to pressure overload [41]. On the other hand, other evidence [64, 65] showed that both Nox2 and Nox4 contribute to the increase in ROS and injury by ischemia/reperfusion. However, low levels of ROS produced by either Nox2 or Nox4 regulate HIF-1*α* and PPAR-*α*, thereby protecting the heart against the ischemia/reperfusion injury, suggesting that Nox also act as a physiological sensor for myocardial adaptation [64, 65].

Higher levels of Nox1 and Nox2 in porcine isolated coronary arteries (PCA) were found in males, although Nox4 was higher in females [66]. It could be possible that higher expression of Nox subunits in men could in part explain why males exhibit higher levels of oxidative stress than females. Wong et al. [66] also showed that the Nox subunits played a role in endothelium-derived hyperpolarization in PCAs of males but not females, which could further show that an increased level of Nox activity is indicative of more oxidative stress in males. Also, ovariectomy of female rats NADPH oxidase activity treatment with 17b-estradiol restored NADPH-oxidase activity to normal levels [63]. These data further suggest that estrogen may contribute to decreased oxidative stress, possibly via the regulation of NADPH oxidase activity especially the Nox1, Nox2, and Nox4 subunits. It has also been suggested that males have higher NADPH oxidase activity levels due to an angiotensin II-mediated mechanism. In endothelial cells, 17b-estradiol receptors (E2-R) signaling exerts anti-inflammatory effects by augmenting eNOS-induced NO signaling [67], attenuating the major cellular source of ROS and NADPH oxidase [40, 68], and inhibiting the expression of endothelial leukocyte adhesion molecules such as vascular cell adhesion molecule 1 (VCAM1) and intercellular cell adhesion molecule (ICAM1) [69]. In cardiomyocytes, E2 stimulates the expression of the prosurvival kinase, Akt, which inhibits cellular apoptosis in women [70] and promotes cell survival [71]. Moreover, E2 attenuates CM remodeling in response to pressure overload [72] and augments myocardial angiogenesis, thereby increasing capillary density [25, 73].

The different response to ROS could partially also explain the difference in the therapy's efficacy. A study on spontaneously hypertensive rats shows that females are less susceptible to angiotensin II-mediated increases in oxidative stress [27]. Another study with wild-type mice showed that superoxide production decreased in male Nox2 knockout mice, but not in females, suggesting that Nox2 plays a role in mediating ROS generation in response to angiotensin II in males but not in females [74]. Another study [75] found that males have higher levels of p47, which is a cytoplasmic subunit of NADPH oxidase. This subunit is connected to the angiotensin II system as angiotensin II binds to the angiotensin type I receptor, which then induces the phosphorylation of p47. Then, there is a translocation of the phosphorylated p47 into the plasma membrane, which is an essential step in the assembly of the NADPH oxidase complex and the initiation of ROS production. This study found that p47 levels did not change with estrogen levels, thus representing an estrogen-independent mechanism, suggesting that regulation of NADPH oxidase activity may be more complex and likely the result of a combination of estrogen-dependent and estrogen-independent mechanisms [75]. Male spontaneously hypertensive rats were found to have higher levels of superoxide generation than female rats [76]. Gender differences associated with NADPH oxidase activity are responsible for the higher levels of superoxide in males. Male spontaneously hypertensive rats also have lower levels of nitric oxide (NO) due to its degradation by superoxide, thus contributing to oxidative stress [76]. In a study with lipopolysaccharides (LPS) to induce inflammation and oxidative stress [77], it was found that female rats treated with LPS had a greater stroke volume and cardiac output compared to male rats, while male rats with LPS treatment were found to have systolic dysfunction after 24 hours whereas female rats did not [77]. These data reveal a significant sex difference associated with LPS injection and that female rats seem to recover cardiac function faster than male rats. Sun et al. [78] have reported that females have increased levels of eNOS associated with the cardiomyocyte-specific caveolin and that after ischemia/reperfusion, females have increased translocation of nNOS to caveolin-3. Sun et al. [78] further showed that in females showing less ischemia/reperfusion injury than males, females have increased S-nitrosylation of the L-type-calcium channel, which results in decreased calcium entry via the L-type calcium channel. The increase in NOS in females under conditions associated with increased calcium (which activates NOS) results in increased S-nitrosylation of the L-type calcium channel, less calcium entry, and therefore less calcium loading during ischemia. Previous studies [79–81] have shown that reducing calcium levels during ischemia is cardioprotective. Overall, the results of all these studies have demonstrated that there is a clear association between gender, oxidative stress, and HF. The question remains whether these gender differences with oxidative stress explain the gender differences in overarching diseases [82].

Moreover, recently, some studies [83–85] have been carried out to decipher whether a disparity in the response to stress could take place in cells coming from males and females concerning the chromosomal asset. XX and XY cells, independently from their origin (e.g., mice, rats, and humans) and histotype (e.g., muscular and endothelial vessel cells, cardiomyocytes, neurons, fibroblasts), display different responses to stress: XX cells are more prone to autophagic cytoprotection and senescence, whereas XY cells are easier and undergo apoptosis. Under the same stress, e.g., oxidative, a sex disparity can be found in intracellular parameters of importance in cell fate [86]. This has been attributed to sex-biased miRNAs, which are regulated by estradiol or are expressed from loci on the X chromosome due to incomplete X chromosome inactivation [25]. Interestingly, whereas estradiol-induced miRNAs predominantly appear to serve protective functions, many X-linked miRNAs have been found to challenge microvascular and myocardial integrity. Thus, menopausal estradiol deficiency, resulting in protective miRNA loss and the augmentation of X-linked miRNA expression, may contribute to the female-specific cardiovascular etiology in HF with near-normal/normal LVEF [25].

## 3. Dietary Phytochemicals and HF

Dietary phytochemicals include a large group of nonnutrient compounds from a wide range of plant-derived foods and chemical classes [87, 88]. Polyphenols are the most commonly studied classes of dietary phytochemicals. They are considered an innovative nutritional strategy that helps to reduce cardiovascular risk factors, with a beneficial role in endothelial function and oxidative stress as demonstrated by experimental and clinical models of cardiovascular impairment [89, 90]. Importantly, investigation on phytochemical effect on cardiac remodeling appears to be promising [91]. Indeed, a large number of experimental models report that phytochemicals acting through the modulation of oxidative stress response to ischemic injury reduce cardiomyocyte apoptosis, necrosis, and cardiac fibrosis [92, 93]. Dietary phytochemicals are commonly present in fruits, vegetables, nuts, spices, coffee chocolate, cacao, and wine. Although these compounds are well presented in nature, some limits characterize their bioavailability: it is affected by food preparation techniques, gastrointestinal metabolism, and microbial flora. Also, data regarding phytochemicals active metabolites in tissues are rare and difficult to apply [94]. In this section, we initially describe the characteristics of polyphenols and phytosterols and their role on oxidative stress and endothelial function, and then we discuss the most relevant findings of their applications in experimental and clinical models of HF.

### 3.1. Polyphenols

#### 3.1.1. Classification

Polyphenols include a large class of natural compounds of plant origin found in fruits, legumes, vegetables, cereals, spices, nuts, olives, chocolate, tea, coffee, beer, and wine [95]. They are water-soluble compounds characterized by one or more phenolic rings classified in flavonoid and nonflavonoids.

Flavonoids chemically consist of two phenyls and one heterocyclic ring and are further subdivided into flavones, flavanols, flavanones, flavonols, isoflavones, and anthocyanidins. Well-known examples of flavonoids are quercetin, catechin, epigallocatechin, genistein, hesperidin, delphinidin, apigenin, and luteolin. Blueberries, parsley, black tea, wine, and cocoa are rich in flavonoids [96, 97].

Nonflavonoids include phenolic acids, stilbenes, and lignans, comprising products like curcumin, resveratrol, tannins, chlorogenic acid, gallic acid, and caffeic acid.

Polyphenols are secondary plant metabolites characterized by astringent color, bitterness, and protection against ultraviolet radiation and pathogens [98]. They are firstly hydrolyzed by intestinal enzymes or by colonic microflora and poorly absorbed. However, it is reported that resveratrol is characterized by a good absorption rate and by a very low bioavailability: 92% of the administered dose was excreted in urine and feces [99]. Their plasma concentration depends also on their ability to bind to metal cations and proteins. Once reaching the colon, they are metabolized by microbiota producing several bacterial metabolites [95]. During phase I metabolism, isoflavones and flavonoids undergo O-demethylation and hydroxylation catalyzed by CYP1A1, CYP1A2, and CYP1B1 [100]. Phase II metabolism is characterized by methylation, glucuronidation, sulfation, and ring-fission metabolism. Catechol-O-methyltransferase (COMT) and sulfotransferase SULT1A1 and SULT1A3 are described as key enzymes involved in this process in mice, rats, and humans [101]. A considerable number of studies [102–104] report that in phase III metabolism, dietary phytochemicals influence ATP-dependent efflux pumps such as multidrug resistance-associated glycoprotein (MGP), p glycoprotein (PGP), and organic anion transporters (OAT) by inhibiting their activity.

### 3.2. Mechanisms on Oxidative Stress

Polyphenols have strong antioxidant properties, and their activity on oxidative stress is determined by their capacity to (a) upregulate antioxidant defenses, (b) scavenge and interact with ROS, and (c) inhibit enzymes involved in ROS generation. Polyphenols interact with the hydrophobic core of the membrane layer avoiding ROS access. In the same way, they affect the oxidation rate of proteins and lipids [105]. Furthermore, structural features of polyphenols, as hydroxyl groups, catechol group on the B-ring, and carbonyl group in the C-ring influence the scavenging of metal ion chelation and free radicals. Indeed, quercetin is characterized by iron-chelating properties [106]. Modification of hydrogen peroxidase, nitric oxide synthases (NOS), cyclooxygenase (COX), and lipoxygenase (LOX) has been demonstrated, and their inhibition significantly reduces the level of key mediators and regulators of proinflammatory response as NO, leukotrienes, and prostaglandins [107].

It has been proposed that an indirect antioxidant enzymatic defense is due to hormetic mechanisms where polyphenols induce an adaptive cellular response to oxidative stress [108–110]. Different investigations have reported that the physiological action of polyphenols on endothelium is related to (a) endothelial NO synthase activity production, (b) increase of eNOS expression, (c) inhibition of endothelin-1 synthesis, (d) attenuation of PGI2 synthesis, and (e) intracellular Ca2+ pathways. Several pioneer studies have reported that polyphenols produced endothelium-dependent vasorelaxation that was generally associated with increased NO release and cyclic GMP formation and blunted by inhibitors of NO synthase, indicating that it was mediated by the NO-cyclic GMP pathway [111]. All these pathways are involved in the HF genesis and progression, suggesting a possible role of dietary phytochemicals in this syndrome.

### 3.3. Polyphenols and HF in Experimental Models

#### 3.3.1. Quercetin

Quercetin is abundant in grains, red onions, apples, and citrus fruits, with antioxidant, anti-inflammatory, and anticlotting properties [112]. In rodent models of hypertension, it has demonstrated a dose-dependent antihypertensive effect [113]. In particular, quercetin treatment reduces vascular production of ROS and was associated with downregulation of NADPH oxidase subunit [114]. It has been reported that, in experimental models of hypertension, quercetin attenuates left ventricular hypertrophy, pressure overload, and isoproterenol-induced cardiac hypertrophy [113, 115, 116]. Then, from these results, it may be hypothesized that there is a plausible effect of quercetin in HFpEF. Indeed, quercetin demonstrated to reduce plasmatic levels of ROS, angiotensin, aldosterone, TGF*β*1 myocardial expression, and collagen deposition [116]. Panchal et al. [117] indicated that quercetin's positive effect on collagen deposition is also related to the inhibition of the NF-*κ*B signaling pathway and the activation of nuclear factor erythroid 2-related factor 2 (Nrf2). Furthermore, in metabolic syndrome experimental models, quercetin showed positive effects on left ventricular relaxation by preventing E/A reduction, mediated by enhancing SOD and glutathione peroxidase activity, inducing Nrf2 nuclear translocation, and increasing ATP myocardial levels [118]. In a mouse model of cardiac toxicity induced by doxorubicin [119], where the modulation of oxidative stress activity was mediated by pAkt pathway, ischemia/reperfusion (I/R) experiments revealed that quercetin improves parameters of left ventricular relaxation and contraction. Another investigation focused on the protective role of quercetin on myocardial I/R injury revealed reduction of infarct size and enhanced myocardial contractility and coronary flow. These results were explained by the role of quercetin on inhibiting the nonhistone DNA-binding protein pathway [120]. Peroxisome proliferator-activated receptor *γ* (PPAR-*γ*) inhibition attenuated the effects of quercetin on ROS production, SOD and glutathione peroxidase activation, suppression of NF-*κ*B pathway, and myocardial apoptosis. These modifications suggested that the underlying protective mechanism of quercetin on myocardial injury is related to the inhibition of the NF-*κ*B pathway by PPAR-*γ* activation. In a study [121], after 10 days of intraperitoneal and oral administration of quercetin, a notable amelioration of LVEF was demonstrated. Other molecules similar to quercetin, such as taxifolin, in both *in vitro* and *in vivo* experimental models of cardiac hypertrophy, have demonstrated significantly reduced ROS production and phosphorylation of MAPK-activated protein kinase 2 and attenuated cardiac fibrosis and left ventricular remodeling [122]. It seems that taxifolin in diabetic cardiomyopathy improves diastolic dysfunction by reducing angiotensin II levels, inhibiting NADPH oxidase activity and inducing JAK/STAT3 activity [123]. The abovementioned results suggest that in HFpEF quercetin and similar molecules may improve left ventricular relaxation at least in part by modulating oxidative stress response which in turn influences cardiac remodeling development. Then, the study of the therapy with quercetin and similar molecules in combination with well-known drugs, as ACE inhibitors/sartans, beta blockers or other modulators of adrenergic system activity, in experimental models of HFrEF would be of interest.

#### 3.3.2. Resveratrol

Resveratrol is a stilbene present in grapes, blueberries, and peanuts produced when plants undergo infections or ultraviolet radiation. Based on the beneficial cardiovascular effect of red wine consumption, many interesting studies [124–126] have investigated the protective mechanism of resveratrol against endothelial dysfunction and myocardial tissue injury. In endothelial cells culture, isolated by bovine aortic rings cultured, red wine extracts increased the intracellular Ca2+ level, enhanced eNOS activity and, as a final result, attenuated NO release [127]. Further studies showed that red wine polyphenols are responsible for the phosphorylation of Akt and eNOS which in turn activate NO release. However, in the presence of PI3 inhibitors, polyphenols failed to induce vasorelaxation, suggesting that the PI3-kinase/Akt pathway is crucial [128]. Both resveratrol pretreatment and treatment in ischemia/reperfusion (I/R) injury has shown to reduce the generation of ROS, to scavenge ROS and to modulate the activity of SOD [129, 130].

Activation of SOD and glutathione peroxidase and reduction of proinflammatory biomarkers as myeloperoxidase by resveratrol were correlated to the activation Nrf2/ARE pathway [131]. Other data indicate that resveratrol reduced the production of TNF-*α*, attenuated the expression of TLR4/NF-*κ*B in the ischemic area, and enhanced the synthesis of NO. Of note, application of sNO inhibitors abolished the positive effects of resveratrol [132]. Moreover, resveratrol significantly improves left ventricular-developed pressure and aortic flow and reduces infarct size area, with a significant amelioration of LVEF [131]. Of interest, a recent meta-analysis performed on preclinical studies concluded that in small animal models of I/R injury resveratrol significantly improves infarct size and the benefits were not affected by the duration of reperfusion [133]. Other pathways involved in the modification of the oxidative stress pathway by resveratrol seems to be attributed to MAPK signaling, Sirt1 and Sirt3 activation, and FoxO/NAD activity. Indeed, experiments [134] have demonstrated that resveratrol induced upregulation of Sirt1 expression and downregulation of FoxO1 and these modifications were associated with restoration of the severe diastolic impairment, inhibition of iron-induced profibrotic effect, and increased expression of myocardial SERCA2 protein. Importantly, the examination of Ca2+ homeostasis, in cardiomyocytes isolated from early iron-overloaded mice, demonstrated elevated diastolic Ca2+ levels and prolongation of Ca2+ decay. These changes were corrected by in vivo SERCA2a gene therapy and resveratrol [134]. The modifications in Sirt1 signaling and oxidative stress response were described also in doxorubicin-induced cardiotoxicity, where resveratrol upregulated the expression of Sirt1 protein by attenuating the acetylation of p53 protein [135]. Other associated findings were reduction of myocardial apoptosis, alleviation of oxidative stress, and reduction of left ventricular end-diastolic pressure [135]. Similar results regarding components of reactive cardiac fibrosis, such as cardiomyocyte apoptosis and collagen accumulation in the extracellular matrix of myocardial tissue, were reported in pressure overload-induced hypertrophy [136]. It should be mentioned that resveratrol can attenuate the transition of cardiac fibroblasts phenotype in myofibroblasts acting through TGF*β*-Smad3 signaling and inhibiting ROS/ERK/TGF*β*/periostin pathway [137]. Riba et al. [126] recently reported that resveratrol treatment showed a protective effect on oxidative stress as indicated by the reduction of iNOS, COX-2 activity, ROS generation. Regarding left ventricular dysfunction, resveratrol-treated rats increased LVEF and reduced left ventricular mass. These beneficial effects were associated with the interaction of resveratrol with cellular survival (e.g., Akt-1, GSK-3*β*) and stress signaling pathways (e.g., p38-MAPK, ERK 1/2, MKP-1) [126, 133]. Therefore, resveratrol administration seems to have high potentials regarding restoration of left ventricular relaxation and reduction of myocardial apoptosis and infarct size area in HF models. The modulation of oxidative stress response appears cardinal. However, further investigation regarding survival and in-depth signaling pathway interactions are necessary.

#### 3.3.3. Curcumin

Curcumin is a polyphenol found in turmeric plants which exhibits anti-inflammatory, antioxidant, and antifibrotic properties. Several studies have tested the hypothesis of a possible cardioprotective role of this compound [138].

Curcumin showed to be effective in inhibiting maladaptive cardiac remodeling and preserving cardiac function after I/R injury. In particular, Wang et al. [139] demonstrated that, after oral administration of curcumin during reperfusion, collagen synthesis was reduced and the expression of TGF*β*1 and phospho-Smad2/3 was downregulated. Moreover, stroke volume, LVEF, and left ventricular end-diastolic volume were significantly improved. Toll-like receptor-2 (TLR2) inhibition by curcumin was also described in I/R experiments, and this effect was associated with preservation of cardiac contractility [140]. It has been proposed that the cardioprotective role of curcumin against myocardial injury is related to the activation of JAK2/STAT3 signaling pathway [141]. Other pathways related to curcumin effects in HF are the modulation of AT1/AT2 receptors and NF-*κ*B [142, 143]. It is also reported that curcumin-reduced acetylation has a beneficial effect against myocardial injury [144].

Curcumin pretreatment in myocardial infarction models induced by left coronary artery ligation attenuated the expression of Sirt1 and reduced collagen deposition. In vitro experiments revealed that the genetic inhibition of Sirt1 by small interfering molecules blocked the effects of curcumin on cardiac fibroblast proliferation and migration [145]. It has been suggested that curcumin cardioprotective role is also related to Sirt1-dependent activation of eNOS. Indeed, in vitro treatment of endothelial cells with curcumin, protected against premature senescence, increased the Sirt1 activation and reduced the production of ROS [146]. Curcumin administration of 5 mg/kg/day and 50 mg/kg/day during 6 weeks in myocardial infarction experimental model resulted in a significant dose-dependent improvement of left ventricular fractional shortening [147]. Recently, the use of curcumin nanoparticles showed promising results regarding cardiac remodeling and antioxidative and anti-inflammatory properties in isoproterenol-induced myocardial infarction model [148]. Therefore, nanotechnology curcumin delivery may be a future application in HF experimental and clinical models.  Table 1 summarizes the abovementioned studies, describing molecular modifications and phytochemical effects on the cardiovascular system.

### 3.4. Polyphenols and HF in Humans

Despite the preclinical studies reporting excellent results of quercetin, curcumin, and resveratrol on infarct size reduction, diastolic function, and cardiac remodeling, the application of these compounds in clinical HF models is limited. Quercetin administration in hypertensive and obese patients initially reported some positive effects in blood pressure control without showing any modulation of oxidative stress or endothelial function [149, 150]. However, other studies failed to show similar results. A recent study reports that quercetin administration for 12 months in patients with gout and hypertension, normalized the blood pressure control and improved left ventricular diastolic function [151]. In a randomized study [152] on patients with stable angina under optimal drug therapy, 120 mg twice/day of quercetin reported amelioration of left ventricular systolic and diastolic function, with a reduction in the circulatory level of proinflammatory cytokines as IL-1*β*, TNF-*α*, and NF-*κ*B [153]. In patients undergoing [154] CABG surgery, the incidence of in-hospital myocardial infarction was decreased from 30.0% in the placebo group to 13.1% in the group treated with 4 g/day of curcuminoid (adjusted hazard ratio 0.35, 0.13 to 0.95, *p* = 0.038) and postoperative C-reactive protein; N-terminal pro-B-type natriuretic peptide levels were also lower in the curcuminoid than in the placebo group. It should be mentioned that the incidence of drug-related adverse events was not different between the placebo and curcuminoid groups, and gastrointestinal symptoms were the main drug-related adverse events.

Although the administration of resveratrol has revealed beneficial anti-inflammatory effects and amelioration of blood pressure control in a different population, no data are available on its effects on left ventricular relaxation or diastolic function [155]. A triple-blind placebo control trial [156], investigating the role of resveratrol and magnesium stearate administration, did not find inflammatory profile reduction. Participants were randomized in homogenous groups for age, gender, comorbidities, cardiovascular risk factor, and LVEF. This study also did not describe changes in diastolic function after resveratrol therapy [156]. In contrast, Milatrau et al. [157] describe not only a reduction of triglycerides and total cholesterol level but also less angina pectoris episodes during and after a short-term follow-up, with a significant reduction of NT-proBNP levels after resveratrol therapy, that could suggest an amelioration of left ventricular function.

Some studies suggest that resveratrol may have an impact on myocardial function in HF patients. Resveratrol administration in a population of myocardial infarction patients [158] resulted in improvement of left ventricular diastolic function, endothelial function measured by flow-mediated vasodilatation, and LDL cholesterol, without improvement in LVEF. Indeed, a positive trend in LVEF amelioration was assessed without achieving a statistical significance (mean LVEF at baseline and after 3-month treatment with 10 mg of resveratrol in placebo and resveratrol group were respectively 52.42 ± 1.55%, 51.33 ± 1.84% and 54.77 ± 1.64%, 55.83 ± 1.94%). Another randomized placebo-controlled study [159], on the effect of flavanol-rich chocolate in HF patients, reported an improvement of endothelial function and inhibition of platelet activation in HF patients. In Table 2, we report studies focused on the phytochemicals use in human HF models.

By these results, it could be speculated that polyphenols might have a more pronounced role in HFpEF than HFrEF. However, in complex, investigation of polyphenol efficacy in patients with cardiovascular disease or cardiovascular risk factors has not reported the same fascinating results as in experimental models. The most updated meta-analysis on this topic [160], in fact, reports that there is no significant amelioration of the lipid, atherosclerotic, and glycemic profile and no significant reduction of blood pressure. It should be mentioned that these studies are characterized by important limitations, as small sample sizes, differences in doses, and protocols. Second, the structural and physiological differences between polyphenols make challenging their short- and long-term health effects. Third, different metabolism of polyphenols can differently affect physiological functions, producing high interindividual differences in the biological response. Indeed, pharmacokinetic studies of polyphenols on humans are very few. Moreover, biotransformation of polyphenols is still debatable and should be considered that drug-metabolizing enzymes in phase I, II, and III represent potential sites of interactions between drugs and polyphenols.

### 3.5. Phytosterols

Phytosterols are bioactive components of plant origin, not synthesized in the human body, that regulate the membrane fluidity of plant cells. The main food sources for phytosterols are vegetable oils, nuts, and cereals. Based on their chemical structure, they are classified in plant sterols and plant stanols. Plant sterols are distinctive for the presence of a double bond in the sterol ring and the most studied sterols are campesterol, sitosterol, and stigmasterol. While examples of plant stanols are campestanol and sitostanol. About 0.5 % of dietary plant stanols and 5% of dietary plant sterols are systemically absorbed [161]. In brief, the absorption process includes their incorporation into mixed micelles, transportation from the intestinal lumen to enterocytes mediated by Niemann Pick C1 Like 1 (NPC1L) protein, and phytosterols secretion into the intestinal lumen mediated by efflux transporters as ATP-binding cassette (ABC) proteins known as ABCG5 and ABCG8 [162].

The speculation with regard to a potential deleterious effect of phytosterols has been largely motivated by the fact that phytosterolemia, a rare autosomal recessive disease, is characterized by a 50-fold increased circulating concentration of plant sterols and may be associated with early atherosclerosis [163]. Several studies have evaluated the association between plasma phytosterol concentration and CVD; however, the results are conflicting. Dietary phytosterol intake increases plasma levels, but increased plasma phytosterols are believed to be a coronary heart disease risk factor. Epidemiological studies have suggested either a direct association between plasma phytosterols and coronary heart disease risk [164] or a null [165, 166] and even inverse association [167]. In the prospective cohort of the Spanish EPIC study [168], plasma concentrations of the main phytosterols, sitosterol, and campesterol appear to be markers of a lower cardiometabolic risk. The study suggests that moderately elevated plasma sitosterol, but not campesterol, might signal individuals with a reduced risk for CHD. However, the authors concluded that they cannot answer the important question of whether circulating phytosterols are proatherogenic, antiatherogenic, or neutral.

Genser et al. [169] published a systematic review and meta-analysis based on 17 studies involving 11,182 individuals and found no evidence of an association between serum concentration of phytosterols and the development of CVD. The authors of this meta-analysis attributed the great divergence in the results of the studies to the different designs of studies and adjustments for potential confounding variables. The results in the study were conflicting because most of them do not take into account potential confounding variables and are provided in different study designs.

Different studies [170–174] have investigated the role of phytosterols on endothelial function and inflammatory profile. Similarly, the arterial stiffness and the flow-mediated dilatation were not modified in hypercholesterolemia or hypercholesterolemia and diabetes [170, 171]. Nonetheless, Gylling et al. [172] have reported beneficial effects on endothelial function and arterial stiffness. Other authors relate the restore of endothelial function to the modification of biomarkers such as E-selectin and plasminogen activator inhibitor 1 (PAI-1) [143, 173]. However, in a setting of dyslipidemic patients, either modification of inflammatory profile or endothelial function was not observed. Again, a meta-analysis study failed to show a modification of the inflammatory biomarkers as mainly C-reactive protein (CRP), after regular intake of phytosterols [174]. Limited data investigated the role of phytosterols on oxidative stress and modification of ROS release after phytosterol consumption. A recently published meta-analysis [175] evaluated the effects of phytosterol consumption in plasma concentrations of liposoluble vitamins and carotenoids. It included 41 randomized clinical trials (*n* = 3,306) with a mean phytosterol intake of 2.5 g/day. In the analyses adjusted for total cholesterol, there was a significant reduction in the concentration of hydrocarbon carotenoids (*β*-carotene, *α*-carotene, and lycopene) and some oxygenated carotenoids (zeaxanthin and cryptoxanthin). In contrast, there was no significant reduction in the concentration of tocopherol, vitamin D, or retinol. A very important finding of this meta-analysis was that the concentration of these substances remained within the normal range, not indicating that the observed reductions could have negative health implications [175]. Yoshida et al. [176] investigated the antioxidant effects of phytosterol and its components, beta-sitosterol, stigmasterol, and campesterol, against lipid peroxidation. It was found that these compounds exerted antioxidant effects on the oxidation of methyl linoleate in solution. Taken together, the findings of the present study showed that phytosterol chemically acts as an antioxidant, a modest radical scavenger, and physically as a stabilizer in the membranes [176]. It is rational to think that lowering the LDL cholesterol level, as one of the major cardiovascular risk factors, may directly influence the risk of developing coronary heart disease and, as a consequence, may reduce the risk from cardiovascular events. However, it should be underlined that studies evaluating the impact of phytosterols and phytostanols on cardiovascular outcome and possible relationship with HF pathophysiology are lacking.

Prospective cohort studies support the beneficial impact of plant-based dietary patterns on incident HF [177–179]. In a study of 38,075 Finnish people over a median of 14.1 years, higher consumption of vegetables was associated with a lower incidence of HF in men, but not in women [180]. Similarly, among 20,900 healthy male physicians in the Physicians' Health Study I, a greater consumption of fruits and vegetables was associated with a decreased risk of HF [181]. In a subset of the Reasons for Geographic and Racial Differences in Stroke (REGARDS) Cohort of 15,569 persons with no coronary artery disease or HF diagnosis patients with closer adherence to the plant-based dietary pattern had a lower risk of incident HF [179]. In a prospective cohort from Sweden of 34,319 women without cardiovascular disease and cancer at initial assessment, after 12.9 years, greater fruit and vegetable consumption was associated with a lower rate of HF [178]. It is hypothesized that HFpEF may be secondary, in part, to diffuse endothelial dysfunction, including that of the myocardial microvasculature, due partly, to increased inflammation [182]. Many plant-based foods are anti-inflammatory and increase NO bioavailability [183], thereby improving vascular health and potentially ameliorating this microvascular dysfunction. The Dietary Approach to Stop Hypertension in “Diastolic” Heart Failure (DASH—DHF) pilot study evaluated the impact of a DASH diet on 13 postmenopausal women with diastolic HF. After 3 weeks, consuming the DASH diet was associated with significant improvements in blood pressure, arterial elasticity, and oxidative stress [184].

## 4. Dietary Phytochemicals and Sex-Gender

As discussed in the previous paragraphs, the redox state and oxidative stress response vary between female and male individuals. It is therefore not surprising that such variability involves also the effects produced by the phytochemicals in them.

This largely depends on the differences existing between women and men in the adsorption, distribution, metabolism, and elimination (i.e., pharmacokinetics) and in the pharmacodynamics of the xenobiotics [185].

In particular, phenolic compounds undergo important processes of storage, transport, and biotransformation [186]. The latter is very important considering that female and male subjects have a different amount of CYP450 isoenzymes and molecules controlling their activity (e.g., constitutive androstane receptor (CAR) expression is greater in women than in men). Also, polymorphisms in some CYP450 genes are associated with variable levels of their products [186]. The sex-gender-based dissimilarities regard also the enzymes belonging to the phase II of metabolism, and this is reasonable for the phytochemicals, largely metabolized by such proteins including UDP-glucuronosyltransferases (UGTs) and sulfatases (SULTs). Then, the renal elimination of the conjugated phenolic compounds is lower in women compared to men [185].

The differences in the kinetics processes might lead to variability in the effects of all xenobiotics, including dietary phytochemicals. However, the data are still scarce in humans in whom the awareness of the method of action of the drugs, as well as of the natural products, is very important given the potential consequences in the treatment and management of several chronic diseases.

Analyzing the results of the Rotterdam study on the protective effects of quercetin against atherosclerosis, some authors [187] proposed that the better response showed by women could be dependent on a better adsorption of quercetin from rutin. This hypothesis was different from that of the Rotterdam study authors who suggested that the higher benefits could be dependent on the women's estrogen activity [188]. Erlund et al. [189] performed a diet-controlled, double-blind, cross-over study to assess the pharmacokinetics of quercetin in healthy volunteers using doses similar to those derivable from the diet. The evaluation in seven women and nine men revealed that quercetin from rutin but not from aglycone was much more bioavailable in women than in men, particularly in those assuming oral contraceptives.

Not only quercetin but also other (poly)phenolic compounds, such as curcumin and resveratrol, seem to have cardiovascular beneficial effects. However, the strongest evidence has been produced in animal models, while in humans the data are still inconclusive [190].

Curcumin has been suggested to improve vascular endothelial function. In this regard, Santos-Parker et al. [191] recently showed that a 12-week curcumin supplementation is able to increase NO bioavailability and decrease oxidative stress thereby improving both resistance and conduit artery endothelial function in healthy middle-aged and older adults. Notably, the authors reported improvements in both sex-genders but at a greater extent in men compared to women [191].

Resveratrol is one of the most studied phytochemicals because of its potential beneficial effects on several pathologies, including CVD. However, contrasting results have been found. As for other molecules, this could be explained by the numerous factors that potentially influence the resveratrol efficacy and tolerability in humans, such as age, lifestyle, dose, administration medium, gut microbiota, and gender. The latter, as well as the other variables, have not yet been sufficiently investigated [192].

A recent meta-analysis, including 18 randomized trials performed in humans, aimed to clarify whether flavonol supplementation could be useful to reduce biomarkers of CVD risk. The main result was that flavonol consumption improved lipid profile, plasma glucose, and blood pressure but in a variable manner, is strongly dependent on individuals' genetic background and health status. Notably, 9 out of 18 trials included both women and men but most of them did not make a sex-gender-based distinction of the results [193].

In women affected by diabetes mellitus type II, chronic supplementation with quercetin significantly reduced systolic blood pressure [194]. Soy product intake was associated with a reduction of diastolic blood pressure in peri- and postmenopausal women and men [195]. Although both in men and women with mild to moderate hypertension soy products reduced blood pressure [196], the incidence of hypertension was lower among men consuming olive oil [197]. Globally, the above data suggest a plausible sex-gender polyphenol effect on endothelial function.

Ostertag et al. investigated the effects of an acute administration of flavan-3-oil-enriched dark chocolate, one of the bioactive compounds contained in the dark chocolate. By analyzing blood and urine samples of both women and men, the authors found that this flavonol exerted beneficial effects in both sex-genders in a different way. In men, flavan-3-oil-enriched dark chocolate significantly decreased P-selectin expression and adenosine diphosphate-induced platelet aggregation, while, in women, decreased thrombin receptor-activating peptide-induced platelet aggregation and increased thrombin receptor-activating peptide-induced fibrinogen binding. Increased collagen/epinephrine-induced ex vivo bleeding time was observed in both men and women. White chocolate significantly decreased adenosine diphosphate-induced platelet P-selectin expression and increased collagen/epinephrine-induced ex vivo bleeding time only in men [198]. In a prospective cohort study, aimed at assessing the CVD mortality in the US population with a different intake of flavonoids, no significant sex-gender-based heterogeneity in the results was observed. However, when the authors analyzed the data separately in men and women, they found that flavone consumption was associated with a lower risk of fatal ischemic heart disease especially among women and with a lower risk of fatal stroke in men [199].

The impact of sex-gender in the cholesterol-lowering effects exerted by phytosterols also represents a debated issue. Some studies found no differences, while others [200] suggested a possible effect of such phytochemicals in reducing LDL-C levels in men but not in women.

It is important to note that, until now, the clinical studies aimed at investigating the effects of phytochemicals by sex-gender are small-scale studies; thus, they are not powered enough to give a conclusive response to a so complex question.

## 5. Dietary Phytochemicals, Sex-Gender, and HF

As previously discussed, the different promising studies suggest a protective role of different phytochemical on heart function. In isolated guinea pig myocytes, polyphenols seem to act differently on intracellular Ca2+ signaling in male and female cells. Lew et al. [201] found fundamental sex differences in the acute actions of the widely consumed isoflavone genistein in guinea pig ventricular myocytes. These differences result in an overall increase in contraction of male myocytes, but little or no change in females. Although genistein inhibited L-type Ca2+ currents in cardiac myocytes from both male and female guinea pigs, a greater percentage inhibition in females was found, suggesting consequent greater cardioprotection of this phytochemical in females than in males.

Stauffer et al. [202] reported that male mice with hypertrophic cardiomyopathy that were fed the traditional soy-based diet deteriorate to severe, dilated cardiomyopathy and more fibrosis, induction of beta-myosin heavy chain, inactivation of glycogen synthase kinase-3beta, and activation of caspase-3. However, simply changing the diet to a milk-based (no soy) diet prevents these phenotypes [202]. Female mice with hypertrophic cardiomyopathy did not exhibit clinical signs of severe cardiac disease on the soy diet, and they were not significantly affected by the dietary change, supporting the hypothesis that in HF some phytochemicals can act in a different sex-oriented way.

Another in vivo study [203] performed on transgenic alpha-myosin heavy chain gene usually fed with polyphenols showed that while female mice increased their cardiac mass and preserved cardiac contractile function; male mice developed thin ventricular walls and had poorly contractile hearts, suggesting a possible role in HF prevention.

However, in all clinical studies, the influence of sex-gender in HF prevention and therapy was not fully studied. In a randomized control trial [157] performed on patients with stable angina undergone to administration with calcium-fructoborate and resveratrol, after 2 months, a significant reduction in BNP levels and inflammatory markers, like PCR, was observed. Of interest, males and females were included in this study but an interaction between gender and BNP or inflammatory response to resveratrol level was not performed. Similarly, in other studies [158, 159], although resveratrol improved the endothelial function measured by brachial artery flow dilatation and the inflammatory profile and the E/A ratio in HF patients, the sex-gender influence was not taken into account as a confounding variable [159].

## 6. Conclusions

While a large body of evidence supports an association between oxidative stress and HF, and phytochemicals are characterized by important antioxidant properties, until now only a few studies were able to demonstrate some beneficial effects of phytochemicals in HF patients. Scavenging of ROS with antioxidants has proved to not affect in modifying patients' prognosis. Surely one of the main problems in the definition of the phytochemicals' efficacy is related to the sex-gender influence. The still unknown different response to the oxidative stress in women and men surely condition the response to phytochemicals administration, especially in HF subjects. Therefore, further studies are needed to better clarify the possible effects of phytochemicals in HF subjects, taking into account the different sex-gender antioxidant response.

## Figures and Tables

**Table 1:** Phytochemical effects on animal models of heart failure.

Experimental model	Phytochemical class	Dose	Treatment	Molecular pathway	Cardiovascular effect	References
Obesity, male rats	Quercetin	2 mg/kg/day 10 weeks	10 weeks	↓ TNF-*α*, ↓ adiponectin	↓ SBP	Rivera L. et al. 2008 [112]
Hypertension, male rats	Quercetin	10 mg/kg	5 weeks	↓ MDA	↓ SBP, improves endothelial function	Duarte J. et al. 2001 [113]
Hypertension, male rats	Quercetin	11 mg/kg	13 weeks	↑ eNOS activity, ↓ NADPH generation, ↓ p47	↓ SBP, improves Endothelial Function	Sanchez M. et al. 2006 [114]
Pressure overload, male rats	Quercetin	5-10 mg/kg day	3 weeks	↓ MDA, ↓ ERK1/2,↓ p38,	↓ HW/BW	Han JJ.et al. 2009 [115]
ISO induced cardiac fibrosis, male rats	Quercetin /rutin	25-50 mg/kg day		↑ SOD, ↓ angiotensin II, ↓ TGF*β*1	↓ HW/BW	Li M. et al. 2013 [116]
Metabolic syndrome, male rats	Quercetin	0.8 mg/kg	8 weeks	↑ Nrf2, ↓ NF-*κ*B	↓ SBP, ↑ LVEF,↑ E/A ratio	Panchal SK. et al. 2012 [117]
Dyslipidemia, male rats	Quercetin /ezetimibe	0.5% w/w	4 weeks	↓ GSH/GSSG ratio, ↑ Nrf2	↑ E/A ratio	Castillo LR. et al. 2018 [118]
Doxorubicin and I/R, male rats	Quercetin	15 mg/kg/day	6 weeks	↑ MMP-2,↑ pAkt	↓ apoptosis, improves LV relaxation	Bartekova M. et al. 2015 [119]
I/R injury, male rats	Quercetin	50 mg/kg/day	5 days	↓ HMGB1/TLR/NF-*κ*B activity	↑ myocardial contractility, ↑ coronary flow,↓ infarct size	Dong LY. et al. 2018 [120]
I/R injury, male mice	Quercetin	250 mg/kg/day	10 days	↑ PPAR-*γ* activation, ↓NF-*κ*B pathway	↑ LVEF, ↑ FS	Liu X. et al. 2016 [121]
I/R injury, male rats	Resveratrol	100 *μ*mol/l	before reperfusion	↑ SOD,↓MDA,↑ Nrf2	↑ LVEF, improves LV relaxation, ↓ infarct size	Cheng L. et al. 2015 [131]
I/R injury, male rats	Resveratrol	100 *μ*mol/l	before reperfusion	↓ TNF-*α*, ↓MPO, ↓NF-*κ*B, ↓ TLR4	↓ infarct size, ↓ myocardial apoptosis	Li J. et al. 2014 [132]
Iron-induced cardiomyopathy, male mice	Resveratrol	320 mg/kg day	14 weeks	Sirt1 upregulation, FOX1 downregulation, ↑ SERCA2a	↑ E/A ratio, improves LV relaxation	Das SK. et al. 2015 [134]
Doxorubicin-induced cardiomyopathy, male mice	Resveratrol	15 mg/kg/day	3 weeks	Sirt1 upregulation, ↓ p53 acetylation	↓ myocardial apoptosis, improves LV relaxation and contractility	Zhang C. et al. 2011 [135]
Pressure overload model, male mice	Resveratrol	10mg/kg/day	4 weeks	↑SOD ↓ HIF-1*α*	↓HW/BW, ↑ LVEF,↑ FS, ↓ cardiac fibrosis	Gupta PK.et al. 2014 [136]
I/R injury, male rats	Curcumin	150 mg/kg	6 weeks	↓ MDA, ↓ TGF*β*/Smad pathway	↓ cardiac fibrosis, ↑ LVEF, FS, SV	Wang NP. et al. 2012 [139]
I/R injury, male rats	Curcumin	300 mg/kg	1 week	↓ TLR2	↑ myocardial contractility	Kim YS. et al. 2012 [140]
I/R injury, male rats	Curcumin	0.25, 0.5, 1 *μ*M	Before reperfusion	Sirt1 upregulation, ↑ SOD, ↓ MDA	↓ infarct size, improves LV contractility	Duan W. et al. 2012 [141]
Angiotensin II-induced cardiac fibrosis, male rats	Curcumin	150 mg/kg	2-4 weeks	↓ TGF*β*/Smad pathway	↓ SBP, ↓ cardiac fibrosis	Pang XF. et al. 2015 [142]
Surgical-induced myocardial infarction, male and female rats	Curcumin	150 mg/kg	4 weeks	↓ NF-*κ*B, ↑ PPAR-*γ*	↓ cardiac fibrosis, ↓ cardiac apoptosis	Lv FH et al. 2016 [143]
Surgical-induced MI, male rats	Curcumin	50 mg/kg/day	7 weeks	↓ p300 HAT activity	↑ FS	Morimoto T. et al. 2008 [144]
Surgical-induced myocardial infarction, male mice	Curcumin	100 mg/kg/day	1 week	Sirt1 upregulation	↓ cardiac necrosis	Xiao J. et al. 2016 [145]
ISO-induced myocardial infarction, male rats	Curcumin nanoparticles	100, 150, 200 mg/kg/day	15 days	↓ TNF-*α*, ↓ IL-6, IL-1*α*, IL-1*β*	↓ cardiac necrosis	Boarescu PM. et al. 2019 [148]

I/R: ischemia reperfusion; ISO: isoproterenol; TNF-*α*: tumor necrosis factor alpha; eNOS: nitric oxide synthase; NADPH: nicotinamide adenine dinucleotide phosphate; MDA: malondialdehyde; SOD: superoxide dismutase; TGF*β*1: transforming growth factor beta 1; NF-*κ*B: nuclear factor kappa beta; GSH/GSSS ratio: reduced glutathione/oxidized glutathione ratio; MMP2: matrix metalloproteinase-2; HMGB1: high mobility group box 1 protein; PPAR-*γ*: peroxisome proliferator-activated receptor gamma; MPO: myeloperoxidase; TLR: toll-like receptor, Sirt1: sirtuin 1; HIF-1*α* : hypoxia-inducible factor 1-alpha; HAT: hystone acetyl transferase; IL-6: interleukin-6; IL-1*α*: interleukin 1 alpha; IL-1*β*: Interleukin 1-beta; SBP: systolic blood pressure; LVEF: left ventricular ejection fraction; HW/BW: heart weight/body weight; FS: fractional shortening; SV: stroke volume.

**Table 2:** Phytochemical effects on human heart failure models.

Overall population	Study	Setting	Phytochemical class	Dose	Treatment	Molecular effects	Cardiovascular effects	References
26 M 15 F	RDBPC	Prehypertension and stage 1 hypertension in overweight or obese subjects	Quercetin	325 mg twice day	4 weeks	No effect on oxidative stress	↓ SBP ↓ DBP	Edwards RL. et al. 2007 [149]
34 M 34 F	RDBPC	Prehypertension and stage 1 hypertension	Quercetin	162 mg/day	6 weeks	No effect on oxidative stress and endothelial function	↓ SBP	Brull V. et al. 2015 [150]
36 M 49 F	RCT	Stable CAD	Quercetin	121 mg twice per day	9 weeks	↓ TNF-*α*, ↓ IL-1*β*, ↓ IL-10	↑ LVEF, ↑ E/A	Chekalina N.et al. 2018 [153]
69 M 61 F	RDBPC	CABG patients	Quercetin	4 g/day	8 days	↓ CRP, MDA, NT-proBNP	↑ LVEF,↓ post-MI CABG	Wongcharoen W. et al. 2012 [154]
64 M 9 F	RTBPC	Stable CAD	Resveratrol	350 mg/day; 700 mg/day	12 months	↓ PAI-1, ↑ adiponectin	Not mentioned	Tome-Carniero J. et al. 2013 [156]
71 M 45 F	RDBPC	Stable angina pectoris	Resveratrol/calcium fructoborate	20 mg/112 mg/day	6 months	↓ hsCRP	↓ NT-proBNP	Militaru C. et al. 2013 [157]
26 M 4 F	RDBPC	Stable CAD	Resveratrol	10 mg/day	3 months	No effect on inflammation	↑ FMD, ↑ E/A	Magyar K. et al. 2012 [158]
17 M 3 F	RCT	Chronic HFrEF	Flavonol-rich chocolate	40 g/day	4 weeks	No effect on oxidative stress	↑ FMD	Flammer EJ. et al. 2012 [159]

M: males; F: females; RDBPC: randomized double-blinded placebo controlled study; RCT: randomized controlled trial; CAD: coronary artery disease; MI: myocardial infarction; CAGB: coronary artery graft bypass; HFrEF: heart failure reduced ejection fraction; TNF-*α* . tumor necrosis factor alpha; CRP: C-reactive protein; hsCRP: high-sensitivity C-reactive protein; MDA: malondialdehyde; PAI-1: plasminogen activator inhibitor-1; NT-proBNP: N-terminal pro b-type natriuretic peptide; SBP: systolic blood pressure; DBP: diastolic blood pressure; LVEF: left ventricular ejection fraction; FMD: flow-mediated dilatation.
